# Bromine Crossover in Operando Analysis of Proton Exchange Membranes in Hydrogen−Bromate Flow Batteries

**DOI:** 10.3390/membranes12080815

**Published:** 2022-08-22

**Authors:** Alexander Modestov, Natalia Kartashova, Roman Pichugov, Mikhail Petrov, Anatoly Antipov, Lilia Abunaeva

**Affiliations:** 1 Frumkin Institute of Physical Chemistry and Electrochemistry, Russian Academy of Sciences, 119071 Moscow, Russia; 2 EMCPS Department, Mendeleev University of Chemical Technology of Russia, 125047 Moscow, Russia

**Keywords:** crossover, bromine, bromate, flow battery, proton exchange membrane

## Abstract

The manuscript deals with the fundamental problem of platinum hydrogen oxidation catalyst poisoning of the hybrid chemical power source based on bromate electroreduction and hydrogen electro-oxidation reactions. The poisoning is caused by the crossover of bromine-containing species through the proton exchange membrane separating compartments of the flow cell. Poisoning results in a drastic decrease in the flow cell performance. This paper describes the results of the direct measurement of bromine-containing species’ crossover through perfluorosulfonic acid membranes of popular vendors in a hydrogen−bromate flow cell and proposes corresponding scenarios for the flow battery charge−discharge operation based on the electrolyte’s control of the pH value. The rate of the crossover of the bromine-containing species through the membrane is found to be inversely proportional to the membrane thickness.

## 1. Introduction

Today, mankind is making progress toward sustainable development by committing to the well-known 17 sustainable development goals (SDGs) [1]. Major 2030 agenda items include the eradication of the climate crisis and universal access to affordable, reliable and sustainable energy. The evident solution at the edge of ecologic and energy concerns is the efficient usage of renewable energy sources, with solar and wind being top priority. Today, due to significant progress in photovoltaic systems and wind turbine technologies, solar and wind energy generation systems have been established all over the world [2]. Nevertheless, the intermittency of these energy sources has resulted in the demand for effective energy storage systems (ESS). The intermittency of renewable energy opens spatial and temporal gaps between the availability of the energy and its consumption by the end-users; thus, valuable electrical energies are difficult to apply continuously and stably [3]. The employment of large-scale ESS may greatly improve the utilization rate and stability of renewable energy. This demand remains a stumbling block on the way to our zero-carbon future, motivating the development of various energy storage methods. Chemical energy storage is one among the most effective energy systems.

Redox flow batteries (RFB) encompass one of the best combinations of efficiency, cost and flexibility due to their module construction offering independent scaling of energy capacity and power. For this reason, RFBs are one of the most tempting technologies for stationary chemical ESS. However, the absolute values of energy and power densities of RFB remain relatively low, especially in comparison with other secondary electrochemical power sources, such as lithium-ion or lead−acid batteries. Recalling that fuel cells (FC) are the prominent leaders in energy capacity and power density values among chemical energy sources, this manuscript is a humble attempt to propose the evident evolution of the modern RFB systems via its hybridization with FC technology to overcome the main RFB drawbacks listed above.

We recently proposed [4,5,6] the hydrogen−bromate flow battery (HBFB), which provides 1300 W h kg^−1^ of theoretical energy density, approximately 12 times higher than that of conventional vanadium redox flow batteries at 113 W h kg^−1^. The high energy capacity value of HBFBs is due to the six-electron reduction of the bromate anion and the extremely high solubility of bromate salts in aqueous solutions (up to 8 M). Moreover, HBFB’s power density takes advantage of the rapid kinetics of bromine/bromide redox couples involved in bromate reduction at the positive electrode via autocatalytical redox mediator catalysis (EC’’ mechanism) [4,7,8,9]. The autocatalytic traits of the latter enable rapid bromide anion accumulation in the vicinity of the electrode, which increases the rate of bromate reduction.

One should note that the toxicity of pure bromine is rather high. However, since the EC’’ mechanism uses bromine only as a redox mediator inside the solution media near the electrodes, this problem can be neglected for the carefully sealed system if the operation is safe enough and the reagents are properly disposed of. One can also recall that this obstacle did not prevent the commercialization of H_2_^−^Br_2_ redox flow batteries.

The absence of the precious metal catalyst at the positive electrode is another advantage of the HBFB system. A Pt catalyst is needed only at the negative electrode, where hydrogen oxidation occurs. Nevertheless, it was shown earlier [5] that under certain conditions, the crossover of bromine-containing species through the cation exchange membrane can rapidly contaminate the Pt hydrogen oxidation catalyst and abruptly collapse the electric current values, turning down the whole system. The greater the thickness of the membrane, the higher the poisoning effect. With the help of the modified Luggin capillary [10], it was shown in [5] that the degree of the platinum electrocatalyst poisoning was determined by the balance between the rates of the bromine species’ supply to the anode and their removal with the liquid water that permeates the membrane. Several optimal scenarios aimed at minimizing the catalyst’s poisoning effect were proposed [5].

Another goal of this work is to formulate an optimal strategy for the HBFB charge−discharge operation based on the ability of the electrolyte to control the pH value. Contrary to the HBFB discharge process where electrolytes at the positive compartment of the HBFB should be acidic to facilitate bromate electroreduction, the charging process should be performed in alkaline media for bromide electro-oxidation.

One of the challenges on the way to the broad use of redox flow batteries as ESS is the transport of active species through the membranes separating the compartments of the positive and negative electrodes [11,12,13,14]. The adverse effects of crossover on the efficiency of the redox flow battery system consist of three basic detriments to performance:

1. Loss of active materials in chemical reactions;
2. Cross-contamination of electrolytes, which necessitates regular corrections of the electrolytes’ compositions;
3. Corrosion and contamination of the electrode catalysts by species penetrating from the opposite compartment.

Crossover of bromine-containing species through Nafion membranes was extensively studied during the development of H_2_/Br_2_ redox flow batteries [14,15]. The decline in the performance of these batteries was ascribed to the loss of activity of the Pt hydrogen oxidation catalyst caused by the crossover of bromine or/and bromide/tribromide anions through Nafion proton exchange membranes [16,17,18,19,20,21,22,23,24,25,26,27]. Bromide adsorption on polycrystalline Pt was studied in [27,28,29]. Bromide adsorption on Pt from 0.1 mM Br + 0.1 M HClO_4_ solution starts at 0.08 V and reaches 80% of the saturation level at 0.2 V vs. SHE [28]. According to [29], bromide adsorption on Pt (100) in 0.1 M HC1O_4_ + 0.1 mM KBr starts at about −0.01 V vs. SHE. The rate of hydrogen oxidation on Pt at 25 mV overvoltage in contact with 48% HBr was found to be lower than that in 4 M H_2_SO_4_ by a factor of five [24]. The detrimental effect of bromide adsorption on the hydrogen oxidation rate and formation of surface oxides on Pt was also shown in [25].

The main goal of the present work is the evaluation of the crossover rate of bromine-containing species from the cathode compartment to the anode compartment of the HBFB flow cell. It was proposed that the bromine-containing species are mostly oxybromine anions [5]. However, the transport of uncharged bromine molecules that diffuse through the fluorocarbon lattice of perfluorosulfonic acid membranes cannot be excluded. In the steady state functioning of the flow cell, the supply of the bromine-containing species to the anode must be balanced by their removal rate. At the high reduction potential of the hydrogen oxidation electrode, any bromine-containing species, evolving at this electrode by the membrane crossover, will be reduced to bromide via the Pt hydrogen oxidation catalyst. In the absence of other cations, except for H^+^, bromide anions can be removed from the anode only as HBr. These species present in the effluent from the anode compartment were trapped in an additional electrochemical cell filled with aqueous electrolytes and detected by measurements of pH change and by quantitative electrochemical oxidation of bromide. The crossover rate of bromine species though cation exchange membranes of different thicknesses and producers is the main subject of the research.

## 2. Materials and Methods’

### 2.1. Flow Cell Design and Materials

**Figure 1:**
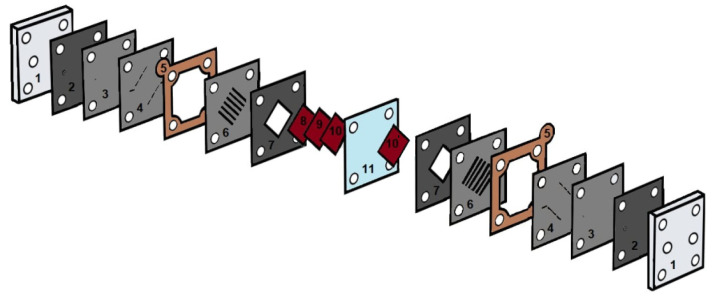
The design of a hydrogen−bromate flow cell: 1—titanium end plates with compression fittings, 2—sealing gaskets, 3, 4, 6—graphite foil plates with the flow fields, 5—a current collector plate made of copper foil, 7—electrode pads, 8, 9, 10—carbon paper cathode; 10′—Pt catalyzed gas diffusion anode, 11—cation exchange membrane.

Design of a hydrogen−bromate flow cell is shown in Figure 1. The design of it was discussed in [30]. Membrane electrode assembly (MEA) of a 4 cm^2^ working area was used in the experiment. Bromate reduction flow through the electrodes consisted of three layers of unteflonized 370 μm thick Toray carbon paper TGP-H-120 (Toray, Industries, Tokyo, Japan). A hydrogen oxidation electrode with platinum loading of 0.8 mg cm^−2^ was made by spray coating catalyst ink on a Sigracet 39BC (SGL Carbon, Wiesbaden, Germany) gas diffusion layer. Catalyst ink contained Pt 40% on XC-72 carbon (HiSPEC-4000, Johnson-Matthey, London, UK), Nafion 10% dispersion (DuPont, Wilmington, DE, USA) and iso-propanol−H_2_O mixture. The gas diffusion electrode was attached to cation exchange membranes via hot pressing at 130 °C, 40 kg cm^−2^ for 3 min. Nafion (Chemours Company, Wilmington, DE, USA) N211, N212, N115, N117 and N1110 membranes were 25 µm, 50 µm, 125 µm, 175 µm and 250 µm thicknesses, respectively, GP-IEM (Liaoning Grepalofu NewEnergy Co., Ltd., Panjin, China) GP-IEM 102, GP-IEM 103 and GP-IEM 105 membranes were 50 µm, 80 µm and 125 µm thicknesse. A Fumatech (Fuma-tech GmbH, St. Ingbert, Germany) F-950 membrane of 50 µm thickness was used to separate compartments of the flow cell. Flow fields and liquid/gas supply channels were formed inside 1.4-mm-thick grafoil sheets (Unikhimtek, Klimovsk, Russia) by a programmable 20-W fiber laser of 1 μm wavelength. Then, grafoil sheets were densified by Carver press at 400 kg cm^−2^ pressure. Flow fields, MEA and PTFE spacers were clamped by titanium endplates with the use of four stainless steel bolts. Controlled flow of dry gaseous hydrogen (99.999%) to the anode compartment was provided by the hydrogen fuel cell test station G-40 (Hydrogenics, Mississauga, ON, Canada). A 1 M NaBrO_3_^−^1 M H_2_SO_4_ mixture was pumped through the positive electrode compartment at a rate of 1 mL min^−1^ by syringe pump NE-1010. In some experiments, 1 M NaBrO_3_^−^1 M H_2_SO_4_ was circulated between a 50 mL flask and the cathode compartment by a BT600-2J (Longerpump, Hebei, China) peristaltic pump.

### 2.2. Methods

#### 2.2.1. Electrochemical Measurements

**Figure 2:**
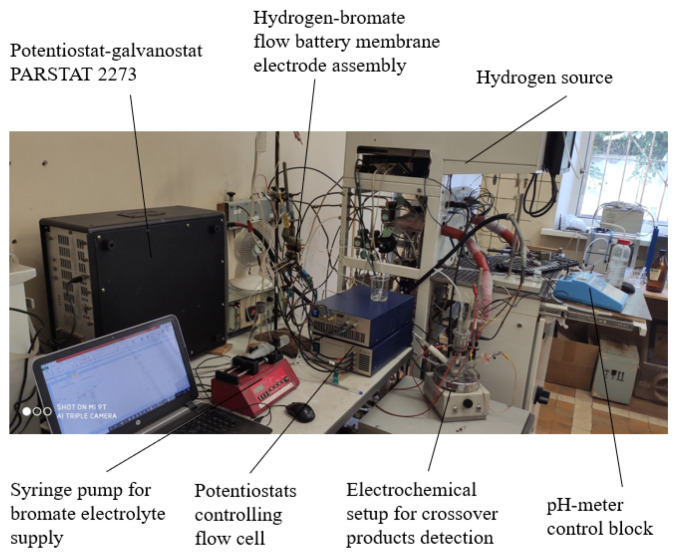
The overall experimental setup for the hydrogen−bromate flow battery study.

Unless otherwise specified, the electrochemical measurements were carried out at temperature of 60 °C in a flow cell of the design described above (see Figure 1). The temperature in the flow cell was controlled using a PID temperature controller, an electric heater and a fan. Electrochemical parameters of the flow cell were monitored using potentiostats: ELINS P-45 (electrochemical impedance measurements and low current voltammetry) and ELINS P-200 (measurements of high current voltammetry), both produced by Electrochemical Instruments, Chernogolovka, Russia. The photograph of the overall experimental setup is shown in Figure 2.

#### 2.2.2. Detection of Bromine-Containing Species in the Effluent from the Anode Compartment

In our experiments, the stoichiometry of the hydrogen supply to the anode was at least four. The flow of unreacted hydrogen removed HBr that was produced by the reduction of bromine-containing species that permeated the membrane. To detect HBr in gases leaving the negative electrode compartment of the flow cell, they were bubbled through 80 mL of a 0.125 M K_2_SO_4_ aqueous electrolyte located in a compartment of the working electrode of the standard three-electrode electrochemical cell with the installed glass electrode of the pH meter. The electrolyte in the working electrode compartment was continuously stirred using a magnetic stirrer. The mercury sulfate reference electrode and platinum counter electrode compartments were separated from the working compartment of the cell by sintered glass diaphragms.

##### Detection of Bromine-Containing Species by pH Measurements

HBr is a strong acid that in relatively low concentrations in water dissociates completely, pKa = −8.08 [31]. Its absorption by the aqueous electrolyte changes the electrolyte’s pH value. The change of pH of the electrolyte over time contained in the working compartment of the three-electrode cell was used to determine the amount of HBr that left the anode compartment of the flow cell with excess hydrogen gas. pH changes over time were monitored by recording the data from the display of the pH meter. We calibrated the pH meter just before the measurements by adding aliquots of HBr into the electrolyte of a working electrode compartment of the three-electrode cell. After calibration the cell was washed and refilled with a new portion of 0.125 M K_2_SO_4_.

##### Detection of the Bromine-Containing Species by Electrochemical Method

The concentration of HBr in the working electrode compartment of the three-electrode electrochemical cell was monitored by the electrochemical oxidation of Br^−^ anion at the Pt working electrode. The electrode was a 1 mm stationary Pt disk, manufactured by soldering platinum wire into a glass tube. The end of this tube with a soldered Pt wire was flat polished. Potentiostat PARSTAT 2273 was used to control the three-electrode cell. Differential pulse voltammetry from the POWERPULSE package of the Potentiostat was employed to determine Br^−^ concentration. Square potential pulses with a height of 30 mV, a duration of 0.1 s., a step duration of 0.2 s. and an average rate of change of potential 15 mV s^−1^ toward positive values of potential were applied. The measurements were performed periodically. Prior to measurements, calibration was made by adding aliquots of HBr to the working electrode compartment of the cell.

#### 2.2.3. Main Chemical Transformations

The overall process for the hydrogen−bromate flow battery based on the EC’’ reaction mechanism is described by the reaction:

3H_2_ + BrO_3_^−^ → Br^−^ + 3H_2_O (1)

It consists of the bromate reduction reaction [32]:

BrO_3_^−^ + 6H^+^ → Br + 3H_2_O − 6e^−^, E^0^ = 1.41 V vs. SHE (2)

and the hydrogen oxidation reaction:

3H_2_ → 6H^+^ + 6e^−^ (3)

**Figure 3:**
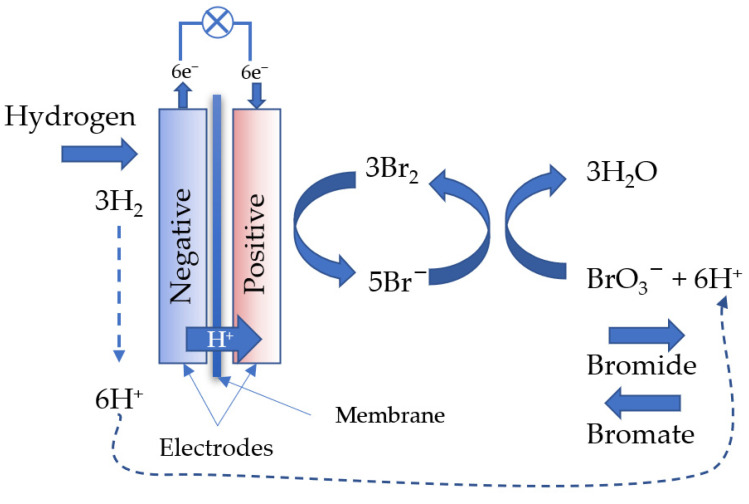
The overall principle of the hydrogen−bromate flow battery operation based on the EC’’ reaction mechanism.

A scheme of this process is shown in Figure 3.

In an acidic medium, the discharge process of the bromate electroreduction consists of two steps. The electrochemical step is a heterogeneous reaction that can proceed even on a low active catalytic surface, such as carbon paper [32]:

3Br_2_ + 6e^−^ → 6Br^−^, E^0^ = 1.087 V vs. SHE. (4)

The chemical step is a homogeneous reaction. It occurs in the bulk solution if the latter is acidic enough:

BrO_3_^−^ + 5Br^−^ + 6H^+^ → 3Br_2_ + 3H_2_O. (5)

During the reduction of bromate to bromide according to reactions (3) and (4), six protons are transferred from the anode compartment to the cathode compartment through the membrane, i.e., the same amount that is consumed in the comproportionation reaction. Therefore, the pH of the catholyte is not changed.

During discharge at the positive electrode, the bromate electrolyte is consumed via reaction cycles (4) and (5), while at the negative electrode, the hydrogen oxidation reaction proceeds at the platinum-catalyzed gas diffusion anode, according to reaction (1B).

It is worth mentioning that in a weakly acidic electrolyte, the formation of a weak hypobromic acid is possible according to the reactions:

BrO_3_^−^ + 2Br^−^ → 3BrO (6)

and

Br_2_ + H_2_O ↔ HOBr + HBr (7)

The standard reduction potential of the hypobromite ion to bromide in an acid medium is 1.341 V, and in an alkaline medium it is 0.766 V [32,33]. Thus, at an electrolyte pH close to neutral, the reduction of hypobromite is possible already at a potential of E = 1.1 V, that is, at a potential close to the potential of bromine’s reduction to bromide.

Recharging the battery requires alkaline media. In alkaline conditions the charge process of bromide anion electro-oxidation occurs via [32]:

Br^−^ + 6OH^−^ → BrO_3_^−^ + 3H_2_O + 6e^−^, E^0^ = 0.584 V vs. SHE. (8)

## 3. Results and Discussion

### 3.1. Crossover through Membranes of Various Types and Thicknesses

The aim of the study was not only to determine the magnitude of the crossover of bromine-containing species through membranes, but also to select a membrane that prevents crossover to a greater extent. Nafion (Chemours company, DE, USA) N211, N212, N115, N117 and N1110 membranes of 25 µm, 50 µm, 125 µm, 175 µm and 250 µm thicknesses, respectively, GP-IEM (Liaoning Grepalofu NewEnergy Co., Ltd., Panjin, China) membranes of 50 µm, 80 µm and 125 µm thicknesses and a Fumatech (Fuma-tech GmbH, St. Ingbert, Germany) membrane of 50 µm thickness were studied at a constant 0.9 V voltage. The voltage of 0.9 V was chosen because thick membranes (N117 and N1110) at lower voltages demonstrated almost complete poisoning of the negative electrode, which was expressed in a sharp current drop almost to zero closely after switching on the constant voltage [5]. The computer recorded the change in the flow cell current over time throughout the experiment. The duration of the experiment ranged from 1.5 to 4.5 h, depending on the thickness of the membrane. The pH values of the 0.125 M K_2_SO_4_ electrolyte in a three-electrode cell were periodically recorded and the bromide anion content in the same cell was measured by differential pulse voltammetry. To control the quality of the cell assembly, the spectra of the electrochemical impedance and current−voltage characteristics of the flow cell were measured before the crossover measurements.

**Figure 4:**
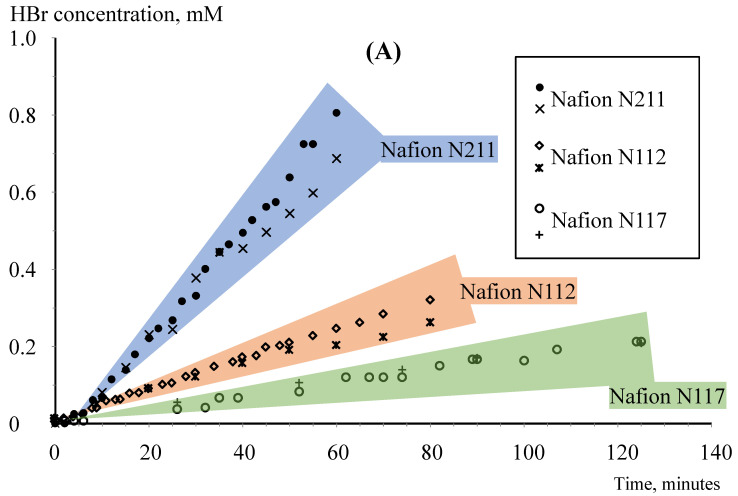

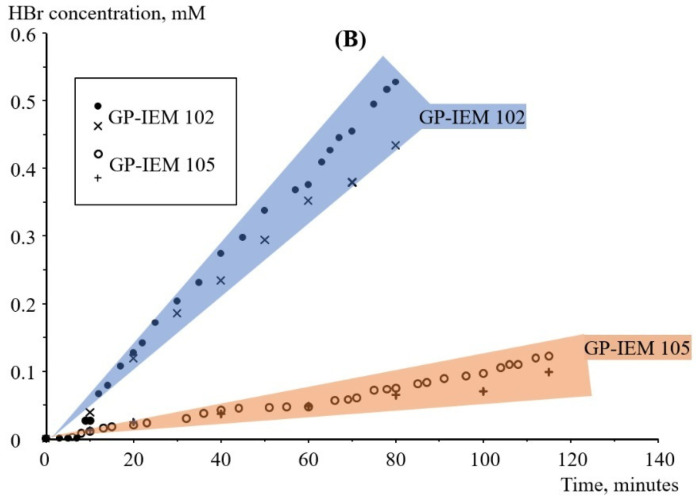
HBr concentration evolution over time in the three-electrode cell while studying the crossover of bromine-containing species in a flow cell at 60 °C with Nafion membranes (**A**) and GP-IEM membranes (**B**). The results obtained by pH measurements are shown by circles and the results of differential pulse voltammetry are shown by crosses.

Figure 4 shows the results of bromine-containing species crossover measurements through Nafion N211, N212 and N117 membranes (diagram A) and through GP-IEM membranes with a thickness of 50 µm and 125 µm (diagram B).

In general, the results of the bromide anion concentration obtained by differential pulse voltammetry and pH measurements are in good agreement and fall on one straight line dependent on the bromide anion concentration over time within a time measurement from 40–50 min. However, at longer times, the results of differential pulse voltammetry deviate from those obtained by pH measurements to lower values of HBr concentration. The nature of this deviation has not been fully elucidated. Possibly, the deviation is due to the slow oxidation of the bromide anion by traces of atmospheric oxygen in the three-electrode cell. Nevertheless, oxidation of HBr with oxygen should not greatly affect the pH value of the electrolyte since the oxidation product will also be an acid.

**Figure 5:**
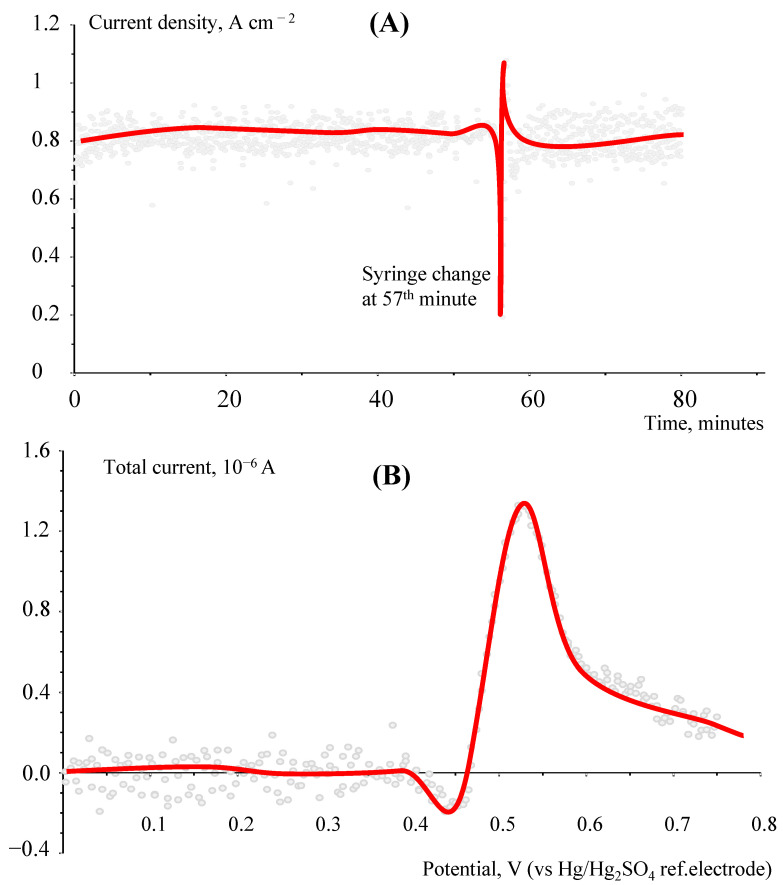
Crossover study through the N212 membrane for the solution of 1 M NaBrO_3_ in 1 M H_2_SO_4_ pumped at a 1 mL min^−1^ rate at constant temperature of 60 °C and voltage of 0.9 V: (**A**) Current density vs. time; (**B**) Potential dependence of current obtained by determining bromide anion by differential pulse voltammetry at the 60th minute of the experiment. For both graphs, the red line is the approximated value of chaotic momentary fluctuations (pale gray points).

Figure 5A shows the example of experimental data of the current density evolution over time obtained with the flow cell with the N212 membrane during the crossover measurement. One can see the discharge current density is practically constant. A sharp fluctuation of the current density at the 57th minute of the experiment is caused by the replacement of the syringe with electrolyte in the syringe pump. Figure 5B shows the dependence of the current on the potential, obtained by determining the bromide anion concentration by differential pulse voltammetry in this experiment. This dependence was measured at the 60th minute of the experiment.

Chaotic fluctuations in the current density (marked as pale grey circles) during the experiments are probably caused by the periodic removal of HBr from the anode compartment of the flow cell as droplets of aqueous HBr. Direct observations have shown that small portions of liquid are periodically passed from the anode compartment through the translucent PFA tube leading to the three-electrode cell. These events were accompanied by changes in the electrolyte pH in the three-electrode cell and current pulsations in the flow cell. The thicker the membrane in the flow cell, the less frequent and more noticeable the release of liquid portions from the anode compartment. This indicates that the removal of HBr from the negative electrode compartment via the gas phase, in the form of HBr vapor, practically does not occur. Pure HBr is a gas at room temperature. Periodic accumulation of liquid in the negative electrode compartment of the flow cell and its release led to temporary blocking of the surface of the hydrogen electrode and fluctuations of the flow cell current. This also shows the importance of water transport through the membrane for the removal of HBr from the anode compartment.

**Figure 6:**
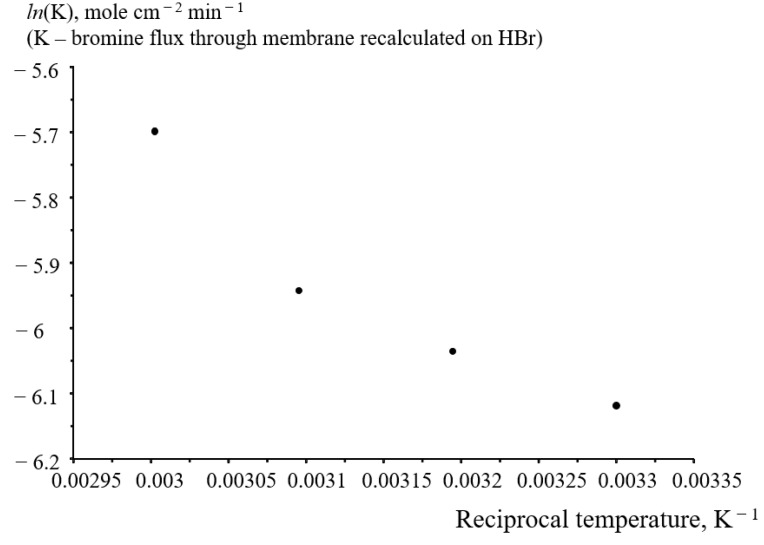
Temperature dependence of the crossover flow of bromine-containing species through the N211 membrane in terms of HBr.

Figure 6 shows an example of the temperature dependence of the crossover rate of bromine-containing species through the N211 membrane in Arrhenius coordinates. The dependence strongly deviates from a straight line, apparently because temperature influences not only the permeability of the membrane for bromine-containing species, but also the membrane thickness, and, probably, the chemical composition of bromine-containing species themselves.

**Figure 7:**
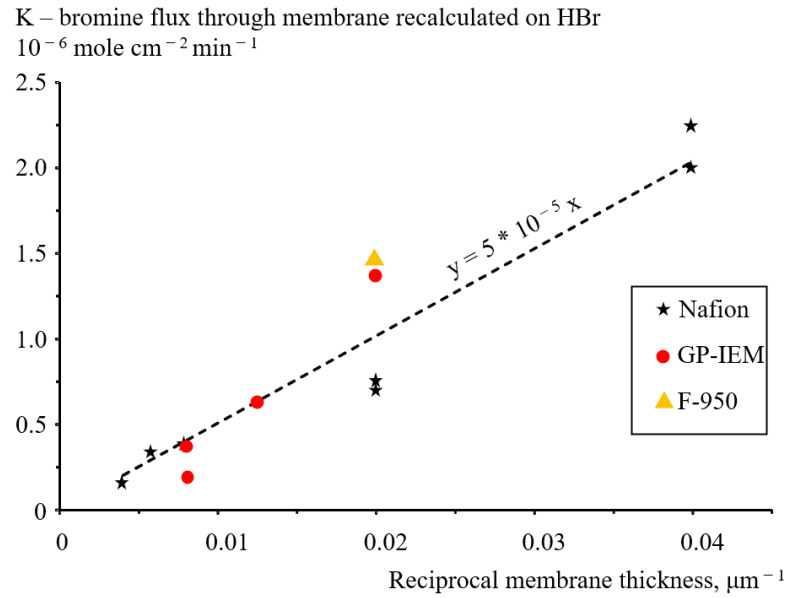
Summarized results of the crossover flux of bromine-containing species recalculated as HBr at a constant temperature (60 °C) and voltage (0.9 V) vs. the reciprocal thickness of the membranes. The dashed line represents empirical linear dependence (9).

The flux of bromine-containing species through a unit (1 cm^2^) of the surface of various membranes under various conditions was calculated in terms of HBr amount from the linear time dependence of the HBr concentration in the three-electrode cell. Figure 7 summarizes these results in the form of the crossover rate vs the reciprocal of the membrane thickness through all the membranes under study. Attention is drawn to the scatter of the crossover values measured on Nafion membranes of various thicknesses. The measurements on the N211 and N212 membranes were duplicated to obtain good reproducibility of the results.

The empirical Formula (9) relates bromine species’ crossover rate with the membrane thickness:

K [mole cm^−2^ min^−1^] = 50 R [µm^−1^], (9)

where R is the reciprocal thickness of the membrane (µm^−1^) and K bromine flux (mole cm^−2^ min^−1^).

### 3.2. Testing of a Hydrogen−Bromate Flow Battery

**Figure 8:**
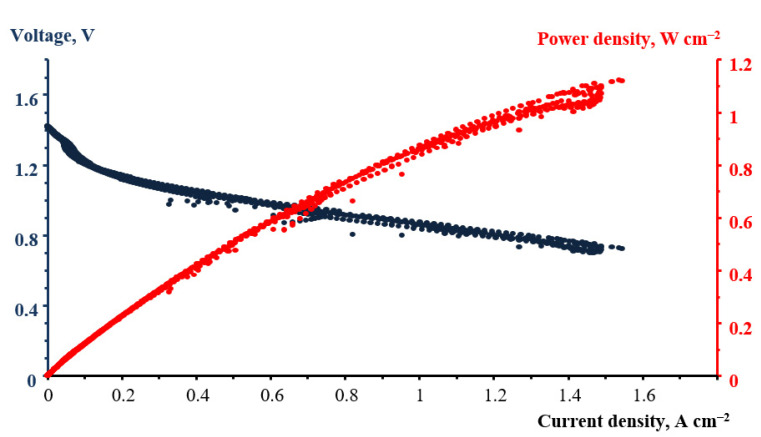
The performance of the flow cell with a N211 membrane.

To preserve the integrity of the data presentation, we describe here the discharge tests of a single cell of a hydrogen−bromate battery during crossover measurements. One should note that these measurements were performed in specially adopted conditions that ensured a reliable measurement of the crossover rate, but these conditions were certainly not optimal for realizing the maximum load characteristics of a flow battery. Therefore, the values of the power density are quite moderate. Figure 8 shows an example of performance curves of the test flow cell with a N211 membrane. At a current density of 1.1 A cm^−2^, flow cell power density reached 1.1 W cm^−2^. The maximum power density measured in similar experiments at 30 °C was only 0.4 W cm^−2^. The set of experiments described below was performed with a circulation of 45 mL 1 M NaBrO_3_ in 1 M H_2_SO_4_ electrolyte through the cathode compartment at a rate ~10 mL/min.

After measuring performance curves of the cell, a potentiostatic mode was set with a voltage of 0.7 V, which approximately corresponds to the maximum specific power demonstrated in Figure 8, and the change in current density with time was recorded.

**Figure 9:**
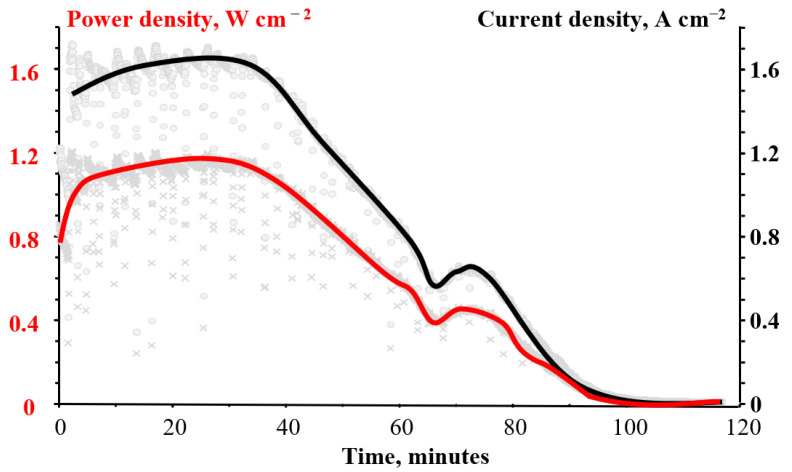
Time example of the variation of current and power densities of a flow cell with a N211 membrane. For both graphs, the solid line is the approximated value of chaotic momentary fluctuations (pale gray points and crosses, correspondingly).

Time variation of current density and power density of the flow cell is shown in Figure 9. For approximately 35 min, the discharge current density was at the level of 1.65 A cm^−2^, and the specific power, respectively, was at the level of 1.15 W cm^−2^. The gradual consumption of bromate in the system leads to a decrease in the discharge current density.

**Figure 10:**
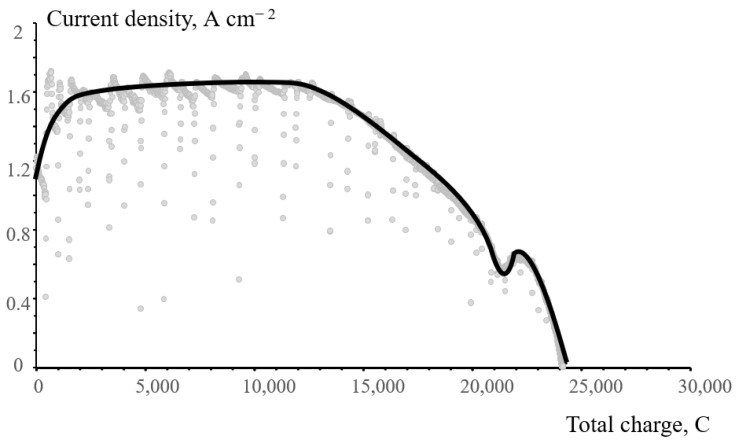
Discharge current density as a function of the total electric charge of a flow cell with a N211 membrane. The solid line is the approximated value of chaotic momentary fluctuations (pale gray points).

Figure 10 shows the change in the discharge current density as a function of the total electric charge that has passed through the cell by a given time for the flow cell with a N211 membrane. According to this figure, the current density decreased to almost zero when the overall charge became equal to 24146 C. This value is close to the stoichiometric charge (26055 C) calculated according to Faraday’s law for the six-electron reduction of 45 mL of 1 M NaBrO_3_. Before long-term experiments, quality checks of the cell assembly were obligatory, therefore a slight discrepancy (7%) in the charge values may be due to some consumption of the bromate electrolyte capacity during these checks.

**Figure 11:**
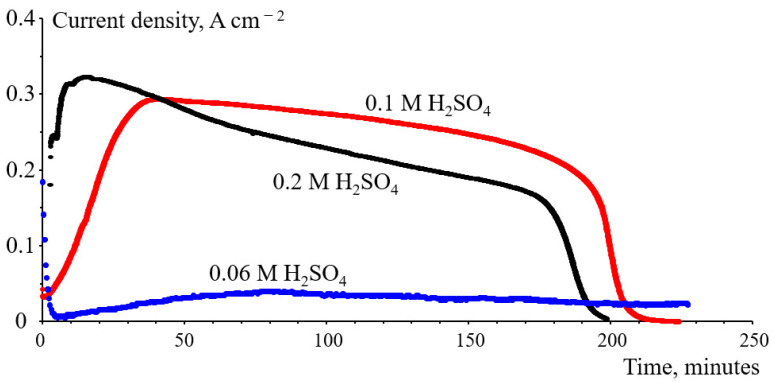
Time variation of current density of a flow cell with a N211 membrane for different concentrations of H_2_SO_4_ in the 1 M NaBrO_3_ catholyte: 0.2 M, 0.1 M and 0.06 M.

As it was shown earlier [6], an increase in acid concentration leads to a sharper increase in the bromate reduction rate. The comparison of the dependences of current density on time measured with different, relatively low concentrations of H_2_SO_4_ in a 1 M NaBrO_3_ catholyte is shown in Figure 11. According to this figure, there is an initial stage during which current density increases until it reaches a maximal value. With the decrease in the concentration of sulfuric acid in a 1 M NaBrO_3_ catholyte from 0.2 M to 0.1 M and 0.06 M, this initial stage of current increase lasts longer: from ~10 min at 0.2 M H_2_SO_4_ to 30 and 70 min at acid concentrations of 0.1 M and 0.06 M, respectively.

Obviously, during this period there is an increase in the concentration of bromine dissolved in the electrolyte due to the acid-catalyzed chemical decomposition of bromate.

Previously, we had obtained high values of the flow battery specific power: 1 W cm^−2^ for the catholyte containing 1 M H_2_SO_4_ and 0.85 W cm^−2^ with 0.25 M H_2_SO_4_ in the catholyte [6].

## 4. Conclusions

The crossover rate of bromine-containing species through the perfluorosulfonic acid membrane separating compartments of HBFB was evaluated by capturing HBr evolving from the anode in a special trap. The change in HBr concentration in the trap over time was measured by monitoring the electrolyte pH change and by periodic electro-oxidation of the bromide anion on the Pt electrode. This method is applicable for measurements of the bromide/bromine crossover rate in H_2_/Br_2_ redox flow batteries, as well.

Considering the relatively large spread of crossover values obtained with different types of perfluorosulfonic acid membranes, we conclude that the membranes studied (Nafion, GP-IEM, Fumatech) do not differ significantly in their permeability to bromine-containing species.

As expected, the crossover rate of bromine-containing species through the perfluorosulfonic acid membranes is inversely proportional to the membrane thickness.

Increase in the flow cell temperature from 30 °C to 60 °C resulted in crossover increase by a factor of only 1.5. Temperature dependence of the crossover rate deviated from the Arrhenius type dependence, apparently, because temperature influences not only the permeability of the membrane for bromine-containing species, but also the membrane thickness, and, probably, the chemical composition of bromine-containing species themselves. On the other hand, the temperature increase from 30 °C to 60 °C resulted in a three-fold increase in the flow cell power density.

It has been shown that the use of a relatively high rate of catholyte pumping and catholyte circulation between the flow cell and reservoir enables achievement of a high power density and nearly 100% utilization of the oxidant.

## Data Availability

Not applicable.
